# Deep sequencing of *SMPD1* gene revealed a heterozygous frameshift mutation (p.Ser192Alafs) in a Palestinian infant with Niemann–Pick disease type A: a case report

**DOI:** 10.1186/s13256-018-1805-x

**Published:** 2018-09-18

**Authors:** Abedelmajeed Nasereddin, Suheir Ereqat

**Affiliations:** 1 Al-Quds Nutrition and Health Research Institute, Faculty of Medicine-Al-Quds University, P.O. Box 20760, Abu-Dis-Esat Jerusalem, Palestine; 2 Genomics Applications Lab, The Core Research Facility, Faculty of Medicine, The Hebrew University, Jerusalem, Israel; 3 Biochemistry and Molecular Biology Department, Faculty of Medicine-Al-Quds University, Abu Dis-East Jerusalem, Palestine

**Keywords:** Sphingomyelinase deficiency, Niemann–Pick disease type A, Mutation, Palestinian child

## Abstract

**Background:**

Niemann–Pick disease is caused by reduced level of the lysosomal enzyme acid sphingomyelinase. Children can survive between 2 and 12 years based on the disease type. Two main types are well known: type A and B. Niemann–Pick disease type A is characterized by severe central nervous system deterioration and hepatosplenomegaly while type B is a progressive hypersplenism accompanied with gradual deterioration of pulmonary function.

**Case presentation:**

We describe an 11-month-old Palestinian baby boy with hepatosplenomegaly, hypotonia, delayed motor development, laryngomalacia, bilateral cherry-red spots, and failure to thrive. Metabolic screening, blood count, differential tests, immunology screen, infectious disease screen, urine, biochemical tests as well as molecular diagnosis were performed. The molecular diagnosis was done by amplifying the whole sphingomyelin phosphodiesterase 1 (*SMPD1*) gene, followed by deep sequencing. The obtained sequences were aligned, *de novo* assembled and compared to human reference gene (GenBank GeneID: NG_011780.1, Ensembl version ENSG00000166311 and protein identified as UniProtKB – P17405).

Two known mutations were identified in our patient: the pathogenic frameshift mutation NM_000543.4(SMPD1):c.573delT (p.Ser192Alafs) and the benign polymorphism NM_000543.4(SMPD1):c.107T>C (p.Val36Ala). The enzyme study showed a very low level of enzymatic activity of acidic sphingomyelinase (0.1 nmol/ml per hour). Correlations between clinical findings, laboratory data, and sequence analysis are presented.

**Conclusions:**

In conclusion, this is the first report about a heterozygote frameshift p.Ser192AlafsX65 in a Palestinian patient with Niemann–Pick disease type A, emphasizing the importance of deep sequencing in genetic diagnosis of this rare inherited disease.

## Background

Niemann–Pick disease (NPD) is an autosomal recessive disease caused by reduced expression of the enzyme acid sphingomyelinase (ASM), encoded by the sphingomyelin phosphodiesterase 1 (*SMPD1*) gene, resulting in lysosomal accumulation of sphingomyelin in [1, 2] the brain causing irreversible neurological damage. NPD types A and B are estimated to affect 1 in 250,000 individuals. Type A is an early-onset neurodegenerative disorder characterized by severe central nervous system deterioration, cherry-red macula, and massive hepatosplenomegaly, leading to death at an early age [3]. Type B is a late-onset non-neuronopathic disease with intermediate clinical presentations that correlate with hepatosplenomegaly and respiratory complications. Most of these cases survive until adulthood [1, 2].

The use of advanced technology, for example, next generation sequencing (NGS), plays a crucial role in sensitive and accurate diagnostic procedures including heterozygote identification, whereas searches for a specific point mutation using Sanger sequencing or enzymatic assays have a limited sensitivity, when compared to NGS. Thus, NGS is recommended for simultaneous mutation detection in multiple exons/introns. Conley and Casanova [4] described the use of NGS in identifying more than 34 new gene defects in autosomal dominant immunodeficiencies with variable penetrance, and revealed *de novo* mutations in disorders with a severe phenotype. This method was a powerful tool in identifying disease-causing gene mutations in different patients.

Recent developments in molecular biology have identified more than 100 mutations in *SMPD1* found in patients with NPD type A/B, which are listed in the Human Gene Mutation Database (HGMD). Most of these mutations are missense (65.4%) or frameshift (19%) mutations. A deletion mutation NM_000543.4(SMPD1):c.1829_1831delGCC (*p*.*Arg610del*) was considered the most frequent reported mutation worldwide, which was associated with an attenuated NPD type B phenotype [5]. Forty mutations were expressed *in vitro* and the impact of the amino acid substitution on the ASM activity has been extensively studied. Twelve mutations retained a residual enzymatic activity higher than 5% of wild type. Eleven of them were found in patients with NPD type B. The p.Phe572Leu retained a residual activity of 30% of wild type. This mutation was found in a patient with NPD type A with compound heterozygosity along with p.Gly247Ser mutation [6, 7]. Six mutations were reported to be associated with type A disease [8–11]. Of them, three mutations were identified in Jewish Ashkenazi patients where the disease is relatively frequent [8–11]. Gluck and colleagues [12] studied 12 Arab Israeli families with NPD type A disease who live in lower Galilee and the West Bank, Palestine. Molecular analysis of these patients identified a novel single base pair (bp) deletion in the *SMPD1* gene 677delT [12]. In 1977 [13], NPD type A was reported in a female Arab infant with no genetic analysis. Here, we describe a high throughput sequencing study of NPD type A of a male Palestinian patient correlated with clinical and biochemical data.

## Case presentation

An 11-month-old Palestinian baby boy presented with distended abdomen, hepatomegaly, and splenomegaly. On evaluation, his body weight was 8.2 kg (third percentile), height 76 cm, (75th percentile), and head circumference 45.8 cm (75th percentile). His parents are first-degree cousins; our patient has three female siblings. All are healthy of Arab Muslim descent, from Seer village-Qalqilya district, Palestine.

Full metabolic screening, blood count, differential tests, immunology screen, infectious disease screen, urine and biochemical tests, as well as amino acid screening were performed as shown in Table 1.

**Table 1:** Blood tests and laboratory analysis

Metabolic test	Unit	Result	Reference
**Sphingomyelinase**	nmol/mL per hour	**0.1**	**≥2.5**
Acid beta-glucosidase		22.8	≥1.8
Acid alpha-glucosidase		8.1	≥3
Galactocerebrosidase		1.4	≥0.4
Alpha-galactosidase		1.5	≥2.8
Alpha-l-iduronidase		9.5	≥2.0
C20 lysophosphatidylcholine	mcg/mL	0.39	≤1.00
C22 lysophosphatidylcholine		0.11	≤0.25
C24 lysophosphatidylcholine		0.11	≤0.30
C26 lysophosphatidylcholine		0.13	≤0.30
**Free carnitine plasma**	**Umol/L**	**28**	**29–43**
**Total carnitine plasma**		**33**	**40–56**
Aspartylglucoseamine urine		Normal	
Alpha mannosidosis urine		Normal	
Fucosidosis		Normal	
GM1 gangliosidosis		Normal	
Sialyloligosaccharide		Normal	
Sialic acid	umol/mmol	86.6	< 95.0
Creatinine	mmol/L	1.69	
**Cholesterol total**	**mmol/L**	**4.84**	**1.15–4.70**
**HDL**		**0.16**	**0.91–2.12**
**LDL**		**3.07**	**≤2.59**
**Triglyceride**		**3.51**	**0.25–0.85**
**Cholesterol/HDL**		**30.25**	**≤4.5**
Amino acids			
**Methionine**	**Umol/L**	**63**	**18–40**
**Threonine**		**216**	**106–164**
**ALK phosphatase**	**IU/L**	**303**	**40–129**
**ALT**		**224**	**≤41**
**AST**		**220**	**≤40**
**CRP**	**mg/L**	**11.25**	**≤5**

Not normal levels are in bold. *ALK* alkaline, *ALT* alanine aminotransferase, *AST* aspartate aminotransferase, *CRP* C-reactive protein, *HDL* high-density lipoprotein, *LDL* low-density lipoprotein

### Deoxyribonucleic acid (DNA) extraction and deep sequence analysis

Genomic DNA was extracted from our patient and his mother’s blood using NucleoSpin® Blood DNA extraction method (MACHEREY-NAGEL, Germany). His father’s blood sample could not be analyzed due to inaccessibility. The entire *SMPD1* gene including the exons and introns (4276 bp) was amplified using LongAmp™ Hot Start *Taq* 2X Master Mix (New England BioLabs) and the two primers SMPD1-P1F: AGAAGGGTAATCGGGTGTCC and SMPD1-P4R: AGCTCCAGGAAAGGAGAAGG (see Zhang *et al*. [14]). These primers were selected among four sets of primers that were previously used to amplify relatively short sequences followed by *de novo* assembly using Geneious bioinformatics software to obtain the full length of *SMPD1* gene [14]. The polymerase chain reaction (PCR) was performed as follows: 35 cycles at 98 °C for 10 seconds, 53 °C for 15 seconds, 72 °C for 50 seconds, then the cycles followed with 72 °C for 5 minutes. The PCR product was visualized on a TapeStation machine (Agilent), cleaned by AMPure XP beads – Beckman Coulter (X0.6), and eluted in 25 μl elution buffer. The product was loaded again on TapeStation (Agilent) to confirm the exact size of the amplified product and cleaning efficiency. The PCR product was quantified by Qubit® Fluorometer (Invitrogen) machine and diluted to 0.2 ng/μl. Finally, 1 ng was used to prepare the next generation library using Nextera XT kit (Illumina) as recommended by the manufacturer. Library purity and quantity were evaluated again by TapeStation and Qubit machines. Concentration of 4 nM was prepared from the two samples. Two million reads for each sample were targeted. Samples were deep sequenced on NextSeq 500/550 machine using the 150-cycle Mid Output Kit (Illumina).

DNA sequences were *de novo* assembled and aligned to human reference gene (GenBank; GeneID NG_011780.1, Ensembl version ENSG00000166311 and protein identified as UniProtKB – P17405) using Geneious bioinformatics software (Biomatters Ltd., Auckland, 1010, New Zealand). Multiple sequence alignment was done online (http://multalin.toulouse.inra.fr/multalin/) as described by Corpet [15].

### Clinical examination and laboratory findings

Due to fever and cough, X-ray imaging of our patient’s chest was done, and was normal. An ultrasound test showed that his liver was 12.1 cm (upper limit for normal 10 cm) with spleen 8.3 cm (upper limit for normal 8.0 cm). No lymphadenopathy was observed. The differential diagnosis for mild hepatosplenomegaly with no lymphadenopathy might underlie a metabolic or hematological disorder. An ophthalmic examination revealed a cherry-red spot in the macula in both eyes.

Complete blood count, differential test, and coagulation test were normal. Microbiology blood culture was negative. Moreover, all tests for Epstein–Barr virus (EBV), cytomegalovirus (CMV), hepatitis (A–C), rubella, *Toxoplasma*, visceral leishmaniasis, pediatric respiratory panel, and anti-tissue glutamines were negatives.

Urine analysis was normal. Molecular microbiology showed negative results for EBV and CMV by quantitative PCR.

Hyperlipidemia was evident, that is, high total cholesterol, low-density lipoprotein (LDL), and triglycerides, while high-density lipoprotein (HDL; 0.16 mmol/L) showed a lower level than normal (Table 1). Cholesterol/HDL ratio (30.25) was significantly high compared to the normal ratio (≤ 4.5).

Amino acid screening showed high level of methionine (63 Umol/L) and threonine (216 Umol/L). Serum alkaline (ALK) phosphatase, alanine aminotransferase (ALT), aspartate aminotransferase (AST), and C-reactive protein (CRP) were above normal limits (Table 1).

### Metabolic screening

Several metabolic enzymes were tested and shown to be normal (Table 1). The alpha-galactosidase was slightly low but not in disease range.

Physiological oligosaccharide in urine does not evoke an oligosaccharidosis (Table 1). A sialic acid assay was conducted, and no increase of free N-acetylneuraminic acid (NANA) storage or excretion was noted, thus, Salla disease was excluded. Free and total plasma carnitine was examined, with slight decrease in free and total carnitine noticed. Sphingomyelinase activity showed remarkable reduction of 0.1 nmol/ml per hour (reference, > 2.5 nmol/ml per hour). Based on clinical findings and laboratory tests, our patient was diagnosed as having NPD. Hence, genetic analysis of *SMPD1* gene sequence was needed to ascertain the pathogenic mutations underlying the molecular basis of this disease.

### Deep sequencing of the whole *SMPD1* gene

**Fig. 1:**
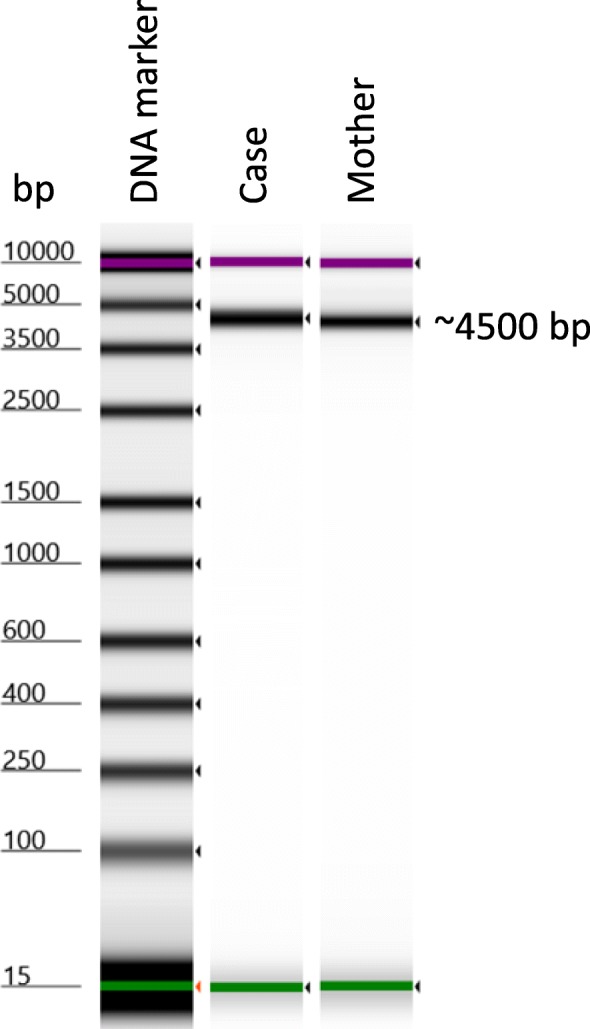
Assessment of DNA size by TapeStation machine. The DNA molecular marker was included; the upper band (*in purple*, 10 kilo base pairs) and lower band (*in green*, 25 base pairs) are indicated. *bp* base pairs

As expected, the amplified product of the two DNA samples (from our patient and his mother) showed bands of approximately 4276 bp using the two primers SMPD1-P1F and SMPD1-P4R (Fig. 1). The obtained DNA sequences were aligned, *de novo* assembled, and compared to the published gene sequence (GenBank GeneID: NG_011780.1, Ensembl version ENSG00000166311 and protein identified as UniProtKB – P17405). A DNA sequence of 4225 and 4229 bp was obtained from mother and patient samples, respectively. The whole gene showed depth of >3000X.

**Fig. 2:**
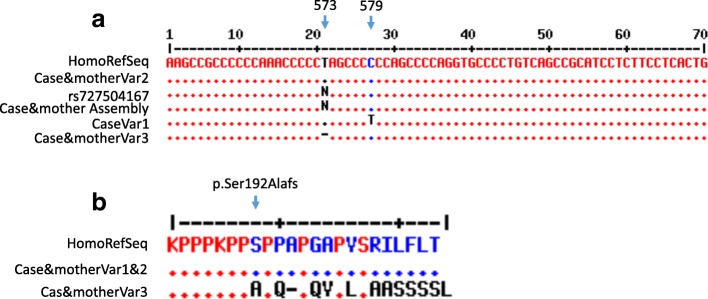
**a** DNA sequence alignment. HomoRefSeq: the human reference gene sequence; rs727504167, the GenBank single nucleotide polymorphism reference rs727504167 in the database (pathogenic allele). Three sequence variants were identified for the patient and his mother. The *arrows* indicate the position of nucleotide substitution/deletion. **b** Aligned sequences of amino acid residues, p.Ser192Alafs causes a frameshift leading to a premature stop codon

**Fig. 3:**
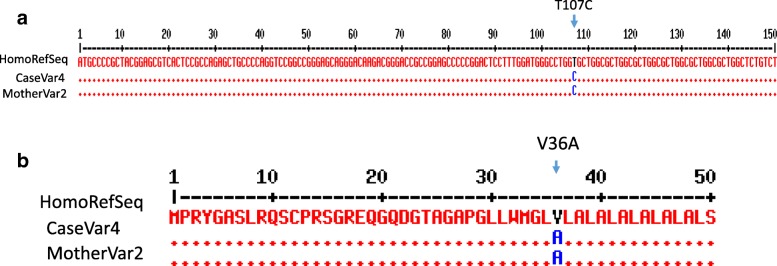
Partial DNA sequence alignment of exon 1 showing (T>C) mutation in the patient and his mother (**a**) and its corresponding amino residue alignment showing p.Val36Ala (**b**)

*SMPD1* sequence analysis of the samples of our patient and his mother revealed the same two heterozygous mutations: a deletion of one base, thymidine, in exon 2 at position 573 of the coding sequence (Fig. 2a) and a substitution mutation at position 107 (T>C) in exon 1. The nucleotides were numbered according to reference sequence (GenBank GeneID: NG_011780.1, Ensembl version ENSG00000166311 and protein identified as UniProtKB – P17405). The NGS identified four sequence variants: single nucleotide polymorphism (SNP) 579C/T was detected in variant 1. Variant 2 was shown to be normal and a frameshift c.573delT (NM_000543.4(SMPD1):c.573delT (p.Ser192Alafs) was detected in variant 3 (Fig. 2a and b). The fourth variant was c.107 leading to conversion of valine to alanine at position 36 (NM_000543.4(SMPD1):c.107T>C (p.Val36Ala)). Notably, our patient and his mother shared these two variants as shown in Fig. 3a and b. To rule out the presence of other possible causal *SMPD1* variants, nucleotide sequences of 863 and 408 bp upstream and downstream from the *SMPD1* coding region were amplified using two sets of forward and reverse specific primers, followed by sequencing.

The primer sequences for the upstream and downstream flanking regions were: (smpd5_2F: CTCATCCTTCCGGTCTGTGT,smpd5_2R:GGACACCCGATTACCCTTCT) and (Smpd_3F:AAGGGTGAAAAAGCCCAAAT,Smpd_3RAAAGATCTCCTTGCCCTGCT), respectively. DNA sequence analyses revealed no pathogenic variants in these regions.

## Discussion and conclusions

Most of *SMPD1* mutations are highly heterogeneous and found in compound heterozygosity mainly in single families. Thus, the genotype cannot be easily correlated with the phenotype. However, some assumptions can be made based on functional analysis of single mutants and for recurrent mutations found in homozygosity [5]. NPD type A was previously described in two studies in the Palestinian community. One study did not conduct a genetic investigation [13] whereas the second study reported a novel single nucleotide deletion 677delT in the *SMPD1* gene in patients with NPD type A deriving from 12 Muslim Arab families [12]. These families live in Galilee and in the West Bank regions. In that study, the delT mutation resulted in a frameshift in the open reading frame and a premature termination at codon number 254 rather than the normal 629. This mutation corresponded to complete absence of sphingomyelinase activity explaining the severity of type A phenotype in these patients.

In our case study, there was a combination of hepatomegaly, elevated liver enzymes activity, and high levels of methionine and threonine with no lymphadenopathy underlying metabolic or hematological disorder [16, 17]. However, the reduced enzymatic activity of sphingomyelinase and hyperlipidemia confirmed the diagnosis of metabolic disease.

NGS has become increasingly common and cost-effective. Analysis of sequencing data is becoming crucial in clinical use in order to discover novel variants that may play a role in disease pathology. Applying direct deep sequencing for the *SMPD1* gene with depth of >3000X for each nucleotide of approximately 5 kilo base pairs (kbp) was very efficient in our case. A previous analysis described the use of too many direct Sanger sequencings targeting the exons and the splicing sites of the introns which may increase the chance of DNA sequencing errors, as several rounds of amplifications and sequencing are needed [14].

Here we report on a case with abnormal clinical and laboratory findings of NPD type A in a heterozygous individual who carried only one pathogenic mutation in the *SMPD1* gene (NM_000543.4(SMPD1):c.573delT (p.Ser192Alafs) which is an unusual finding for an autosomal recessive disease. A similar condition was reported by Lee *et al.* [18] showing that some carrier individuals for ASM-deficiency exhibited clinical phenotypes of NPD type A. Moreover, Simonaro *et al*. [19] demonstrated that the *SMPD1* gene is paternally imprinted and is preferentially expressed from the maternal chromosome and thus distinct clinical presentations were correlated with the amount of residual ASM activity expressed from the mutant maternal allele [14] as shown with p.His461Val mutation in patient number 8 in Zhang and colleagues’ [14] study. Although the father's DNA of our case was not evaluated in this study, we predict, based on *SMPD1* analysis of our patient’s mother’s sample, that the extremely deficient ASM (0.1 nmol/ml per hour) might be due to inheritance of a single, severe frameshift mutation (p.Ser192Alafs) on the preferentially expressed maternal chromosome. This mutation was first identified by Gluck and colleagues [12]; however, it has not been reported in the Arab population. This deletion leads to a new reading frame (p.Ser192AlafsX65) due to change of codon serine 192 to alanine creating a premature stop codon at position 65. Thus, loss of normal protein function due to protein truncation is expected. Unfortunately, the frameshift mutation p.Ser192AlafsX65 has not been expressed *in vitro* but its occurrence in homozygosity in patients with NPD type A strongly suggests its severity [5, 6].

The non-synonymous SNP c.107T>C (NM_000543.4(SMPD1):c.107T>C (p.Val36Ala)) was found in our patient and his mother. NM_000543.4(SMPD1):c.107T>C (p.Val36Ala) missense mutation was first reported in two Iranian patients of whom one was proved homozygous and the other heterozygous. In that study, bioinformatics analysis to predict protein stability of homozygous p.Val36Ala mutation caused reduction in ASM stability [20]. However, this non-synonymous SNP (rs1050228) is considered a benign polymorphism based on HGMD and ClinVar data. Another SNP (579C/T, refSNP: rs1477363633) was identified in our patient; however, this SNP did not affect the final amino acid product in the translation process.

The regulatory region upstream of the *SMPD1* coding sequence, which contains putative promoter elements, and nucleotide sequence downstream from *SMPD1* coding region were analyzed. No other pathogenic variants were found, confirming that the (p.Ser192Alafs) mutation is most likely to be responsible for the reduced ASM activity in our patient. Our findings are consistent with previous studies showing a deficiency in ASM enzyme activity in patients with NPD type A. However, *in vitro* studies are still needed to confirm the functional effect of p.Ser192Alafs mutation on catalytic activity, protein stability, and/or expression of *ASM* gene.

In conclusion, this is the first report about a heterozygote frameshift p.Ser192AlafsX65 in a Palestinian patient with NPD type A. The advanced technology used in this study facilitates the definition of disease-related mutations with whole gene coverage saving time and effort. Screening of this mutation in addition to the c.677delT mutation, which was previously reported in Palestinian patients, should be considered in any local NPD preventive program. This is of particular importance to identify heterozygotes in high-risk families since consanguineous marriages are customary practice in our region. Genetic counseling and prenatal diagnosis would be of considerable value for future family planning.

## References

[1] Schuchman EH, Wasserstein MP. Types A and B Niemann-Pick disease. Pediatr Endocrinol Rev. 2016;13(Suppl 1):674–81. 27491215

[2] Schuchman EH, Wasserstein MP. Types A and B Niemann-Pick disease. Best Pract Res Clin Endocrinol Metab. 2015;29(2):237–47. 10.1016/j.beem.2014.10.002 25987176

[3] Schuchman EH, Desnick RJ. Types A and B Niemann-Pick disease. Mol Genet Metab. 2017;120(1–2):27–33. 10.1016/j.ymgme.2016.12.008 PMC5347465 28164782

[4] Conley ME, Casanova JL. Discovery of single-gene inborn errors of immunity by next generation sequencing. Curr Opin Immunol. 2014;30:17–23. 10.1016/j.coi.2014.05.004 PMC4198453 24886697

[5] Zampieri S, *et al. SMPD1* mutation update: database and comprehensive analysis of published and novel variants. Hum Mutat. 2016;37(2):139–47. 10.1002/humu.22923 26499107

[6] Dardis A, *et al.* Functional *in vitro* characterization of 14 *SMPD1* mutations identified in Italian patients affected by Niemann Pick type B disease. Hum Mutat. 2005;26(2):164. 10.1002/humu.9353 16010684

[7] Toth B, *et al.* Molecular genetic characterization of novel sphingomyelin phosphodiesterase 1 mutations causing Niemann–Pick disease. JIMD Rep. 2012;3:125–9. 10.1007/8904_2011_80 PMC3509870 23430884

[8] Levran O, Desnick RJ, Schuchman EH. Type A Niemann-Pick disease: a frameshift mutation in the acid sphingomyelinase gene (fsP330) occurs in Ashkenazi Jewish patients. Hum Mutat. 1993;2(4):317–9. 10.1002/humu.1380020414 8401540

[9] Levran O, Desnick RJ, Schuchman EH. Identification and expression of a common missense mutation (L302P) in the acid sphingomyelinase gene of Ashkenazi Jewish type A Niemann-Pick disease patients. Blood. 1992;80(8):2081–7. 1391960

[10] Levran O, Desnick RJ, Schuchman EH. Niemann-Pick type B disease. Identification of a single codon deletion in the acid sphingomyelinase gene and genotype/phenotype correlations in type A and B patients. J Clin Invest. 1991;88(3):806–10. 10.1172/JCI115380 PMC295465 1885770

[11] Levran O, Desnick RJ, Schuchman EH. Niemann-Pick disease: a frequent missense mutation in the acid sphingomyelinase gene of Ashkenazi Jewish type A and B patients. Proc Natl Acad Sci U S A. 1991;88(9):3748–52. 10.1073/pnas.88.9.3748 PMC51530 2023926

[12] Gluck I, *et al.* Niemann Pick disease type A in Israeli Arabs: 677delT, a common novel single mutation. Mutations in brief no. 161. Online. Hum Mutat. 1998;12(2):136. 10.1002/(SICI)1098-1004(1998)12:2<136::AID-HUMU11>3.0.CO;2-0 10694919

[13] Zelikovic I, *et al.* Type A Niemann-Pick disease in a female Arab infant. Harefuah. 1977;93(9):239–42. 598759

[14] Zhang H, *et al.* Identification of a distinct mutation spectrum in the *SMPD1* gene of Chinese patients with acid sphingomyelinase-deficient Niemann-Pick disease. Orphanet J Rare Dis. 2013;8:15. 10.1186/1750-1172-8-15 PMC3566977 23356216

[15] Corpet F. Multiple sequence alignment with hierarchical clustering. Nucleic Acids Res. 1988;16(22):10881–90. 10.1093/nar/16.22.10881 PMC338945 2849754

[16] Grasko Y, *et al.* A novel missense *SMPD1* gene mutation, T460P, and clinical findings in a patient with Niemann-Pick disease type B presenting to a lipid disorders clinic. Ann Clin Biochem. 2014;51(Pt 5):615–8. 10.1177/0004563214527067 24643943

[17] McGovern MM, *et al.* Lipid abnormalities in children with types A and B Niemann Pick disease. J Pediatr. 2004;145(1):77–81. 10.1016/j.jpeds.2004.02.048 15238911

[18] Lee CY, *et al.* Compound heterozygosity at the sphingomyelin phosphodiesterase-1 (*SMPD1*) gene is associated with low HDL cholesterol. Hum Genet. 2003;112(5–6):552–62. 10.1007/s00439-002-0893-1 12607113

[19] Simonaro CM, *et al.* Imprinting at the *SMPD1* locus: implications for acid sphingomyelinase-deficient Niemann-Pick disease. Am J Hum Genet. 2006;78(5):865–70. 10.1086/503750 PMC1474038 16642440

[20] Manshadi MD, *et al.* Four novel p.N385K, p.V36A, c.1033-1034insT and c.1417-1418delCT mutations in the sphingomyelin Phosphodiesterase 1 (*SMPD1*) gene in patients with types A and B Niemann-Pick disease (NPD). Int J Mol Sci. 2015;16(4):6668–76. 10.3390/ijms16046668 PMC4424982 25811928

